# Leisure and Productivity in Older Adults with Cancer: A Systematic Review

**DOI:** 10.1155/2021/8886193

**Published:** 2021-04-03

**Authors:** Cynthia Engels, Robin Bairet, Florence Canoui-Poitrine, Marie Laurent

**Affiliations:** ^1^Univ Paris Est Creteil, INSERM, IMRB, F-94010 Creteil, France; ^2^Univ Paris Est Creteil, Faculty of Health, Creteil, France; ^3^School of Rehabilitation, Faculty of Medicine, Université de Montréal, Montreal, Quebec, Canada; ^4^AP-HP, Hôpitaux Henri-Mondor, Département de Santé Publique, F-94010 Creteil, France; ^5^AP-HP, Hôpitaux Henri-Mondor, Service de Médecine et Gériatrie, Unité de Coordination Onco Gériatrie Paris-Sud-Val-de-Marne, F-94010 Creteil, France

## Abstract

**Introduction:**

Self-care, leisure, and productivity are important occupational domains for older adults' quality of life, which might be affected by cancer and its treatment. A great number of publications about older adults focus on function or self-care, so we aimed to analyse how cancer and its treatments affect leisure and productivity. Secondary objectives were to identify whether particular clinical and/or sociodemographic factors were associated with occupational disruptions and to assess the impact of rehabilitation approaches on leisure and productivity in this population.

**Methods:**

A systematic review of the 2009-2019 literature performed on Medline, Embase, and the Cochrane Central Register of Controlled Trials.

**Results:**

1471 publications were retrieved: 48 full texts were assessed; seven of these (four cross-sectional studies, two cohort studies, and a case report) were reviewed, including data on 16668 people (12649 healthy controls, 3918 cancer survivors, and 101 ill patients). Older adults with comorbidities and a low level of activity before cancer diagnosis may be more at risk of occupational disruptions. However, studies focused more on physical activity than leisure and productivity. Two studies mentioned occupational therapy. *Discussion*. As cancer can become a chronic disease, it appears important to also offer occupation-centred assessments and follow-up.

**Conclusion:**

An occupation-centred approach could be developed; its effectiveness must be assessed.

## 1. Introduction

Cancer is a life-threatening illness that is expected to affect 2.7 million older people (i.e., those aged 65 and over) in 2030 [1]. The symptoms of cancer and its treatments (such as fatigue and pain) can limit activities of daily living [2, 3], especially among older adults, who may be affected by frailty and comorbidities and thus are at greater risk of functional limitations [4].

With a view to improving quality of life, the goal of occupational therapy is to promote engagement in occupations. *Occupations* referred to “the everyday activities that people do as individuals, in families and with communities to occupy time and bring meaning and purpose to life. Occupations include things people need to, want to, and are expected to do” [5]. The Canadian Model of Occupational Performance and Engagement (CMOP-E) is a client-centredness conceptual framework concerned with the relationship between occupation, health, and well-being and considering clients as active participants and decision-makers in their therapy [6]. The CMOP-E also allows the study of one's occupations in dynamic interactions with the clients' characteristics and environment and defines occupations in three domains [7]: self-care, productivity, and leisure. Self-care has been described as the activities “that the individual performs for the purpose of maintaining the self in a condition that allows for function” [8]; these include hygiene and dressing, for example. Productivity covers “activities that customarily fill the bulk of one's day and which contribute to economic preservation, home and family maintenance, and service or personal development” [8]; they comprise household management, paid or unpaid work, and academic activities. Lastly, leisure activities are those “that one engages in when one is freed from the obligation to be productive” [8] and can be subdivided in quiet leisure, active leisure, and socialization. So regarding those definitions, the panel of occupations that can be integrated to each category is very broad and changing depending on each individual, his/her environment, culture, personal characteristics, etc. The CMOP-E and its evaluation tool, the Canadian Occupational Performance Measure (COPM), have been previously shown to be useful to identify and address occupational performance issues of adolescents and young adults with cancer [9], as well as with older adults [10–14].

The CMOP-E is aimed at the client reaching occupational engagement, which is usually possible when there is a variety of occupations among the three occupational domains. Indeed, the literature data on healthy older people have notably emphasized the importance of leisure and productivity: for example, previous studies showed that 22% of French people aged ≥50 engaged in quiet leisure more than once a week [15] and that French people aged 65 and above spent more than six hours a day in leisure [16]. Another study showed that 73% of older adults aged ≥60 in the UK declared to engage in leisure occupations, mostly active leisure (23%), with positive feelings about it [17]. Furthermore, a study about older adult's participation in occupations with beginning functional decline who received home-based services also highlighted perceived importance of both personal care and leisure activities [18]. Further, Källdalen et al. [19] identified the valued activities of 240 85-year-old people in Sweden and found that none of them were self-care activities. In contrast, 34% of the women and 5% of the men were interested in productivity (e.g., household management) and about 90% of men and women were interested in quiet leisure. Finally, another study showed that American older adults could spend up to 1878 hours per year in mean in volunteering, with positive outcomes on quality of life [20], while in Sweden older workers over the age of 65 looked for a “harmonious mix of occupations.”

Considering that all three occupational fields contribute to older adults' quality of life and regarding the potential functional consequences of cancer and its treatments, we can hypothesize that the three occupational domains might be affected by cancer and/or its treatments. Therefore, promoting engagement in occupations for people with cancer—as recommended by Occupational Therapy Australia's position statement [21]—should consider the three occupational domains. However, although we found a greater number of quality studies about older people with cancer focusing on function or self-care, we found it difficult to gather information on leisure and productivity, with an occupation-centred approach.

Hence, the primary objective of this systematic review was to determine how cancer disease and its treatments affect leisure and productivity in older adults. The secondary objectives were to build up profiles of older adults with cancer whose participation in leisure and productivity activities is affected and to determine which types of rehabilitation approach have an impact on participation in leisure and productivity activities by this population.

## 2. Materials and Methods

### 2.1. Study Design

We performed a systematic review of the scientific and health literature about leisure and productivity in older adults with cancer, regarding the eligibility criteria and search strategy defined hereafter. The results of the review were reported in compliance with the Preferred Reporting Items for Systematic Reviews and Meta-Analysis (PRISMA) guidelines [22], and the study protocol was registered in the PROSPERO database (to enable PROSPERO to focus on COVID-19 registrations during the 2020 pandemic, this registration record was automatically published exactly as submitted; it has not been checked for eligibility or for sense by the PROSPERO team; registration number CRD42020099857).

### 2.2. Eligibility Criteria for Study Inclusion

Studies were eligible for inclusion if they met the criteria established by the following PICOS framework:

1. Participants: studies included participants aged 65 or over, living or having experienced living with cancer (“survivors”)
2. Interventions: these are all types of interventions regarding leisure or productivity. We referred to the definitions given in the introduction for each category and used the COPM booklet, carefully referring to the specific examples of each type of occupations [23]
3. Comparison: we included comparisons between older people living with cancer and older healthy controls, comparison between the types of cancer, comparisons between the types of cancer treatments, and comparisons between before and after cancer diagnosis. We also included descriptive studies (i.e., with no comparison group)
4. Outcomes: these are leisure or productivity activities, either as individual outcomes or as embedded in quality of life endpoints
5. Study design: we included original publications describing randomized controlled clinical trials, nonrandomized controlled clinical trials, case-control studies, cohort studies, systematic reviews, and meta-analysis, grade I to IV level of evidence [24]. Since we expected to find only a few such publications, we decided to also include case series and case reports, level V of evidence. We excluded editorials, position/statement/opinion papers, conference abstracts, and posters

### 2.3. Search Strategy

Relevant publications between January 2009 and March 2019 were identified, in the following three online databases as recommended in the Cochrane Handbook for Systematic Reviews of Interventions [25]: Medline, Embase, and the Cochrane Central Register of Controlled Trials (CENTRAL). A specific search algorithm was formulated for each database, using the following strategy: ((household OR grandchild∗OR “productive activities” OR productivity OR leisure OR hobby OR hobbies OR recreation OR sport∗OR socialization OR travel∗OR “paper work”) AND (cancer OR oncology) AND (old∗OR elderly OR ageing OR geriatrics) NOT (adolescent NOT paediatr∗NOT pediatr∗)).

The search was limited to full publications in English or French.

### 2.4. Study Selection

Two reviewers (CE and RB, both occupational therapists) independently assessed the list of publications generated in response to the search query. The first selection was based on the titles and abstracts, by considering the criteria defined above in the PICOS section. Disagreements about inclusions were resolved by consensus. The two reviewers then examined full-text versions of the selected publications and independently decided which ones to include in the review. Again, disagreements about inclusion were resolved by consensus.

### 2.5. Data Extraction and Quality Assessment

The following data were extracted from the selected publications and collated in a Microsoft Excel® spreadsheet: authors, year of publication, title, journal, country, aim of the study, type of intervention (if applicable), outcomes, sample characteristics (mean age, sex ratio, cancer site or type, and treatment), measures linked to leisure or productivity (if applicable), study design, and risks of bias.

The risk of bias was assessed by applying the Newcastle–Ottawa Scale (NOS) to cohort studies [26] and the Adapted Newcastle–Ottawa Scale for Evaluating Cross-Sectional/Survey Studies (adapted NOS) to the cross-sectional studies and the case report [27]. Data were extracted, and study quality was assessed independently by the two reviewers. The two assessments were then compared, in order to reach a consensus.

## 3. Results

### 3.1. Literature Search and Selection

**Figure 1:**
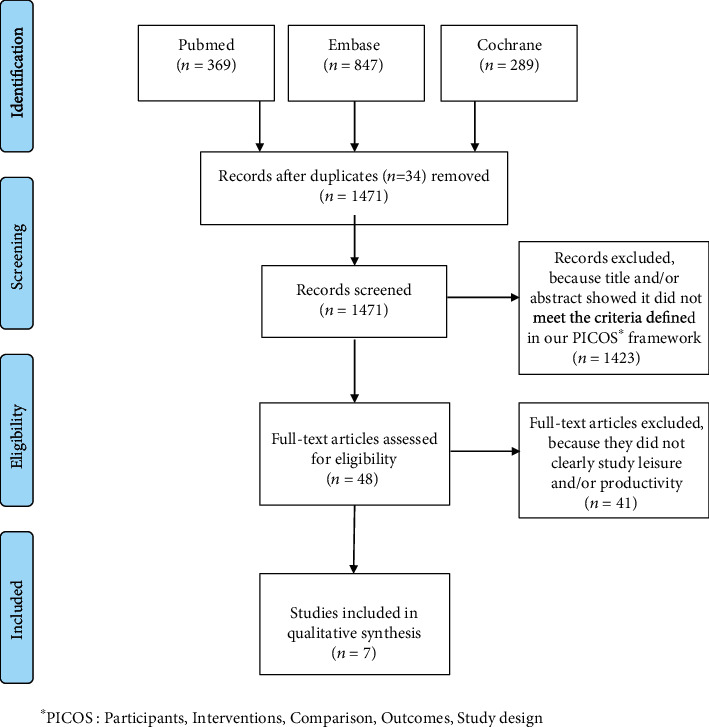
PRISMA flow diagram. ^∗^PICOS: Participants, Interventions, Comparison, Outcomes, Study design. From [22].

Our literature search generated a list of 1505 publications (1471 after the removal of duplicates). After reviewing the titles and abstracts and discussing the disagreements (*n* = 39, mainly with regard to whether leisure and/or productivity activities were covered when only “quality of life” was mentioned), 48 publications were retained for further (full-text) appraisal. Forty-one publications were subsequently excluded because they did not unambiguously study leisure and/or productivity. After disagreements (*n* = 3) had been discussed, seven studies were included in the final review (Figure 1).

### 3.2. Characteristics of Included Studies and Sample Participants

Most of the articles (*n* = 5) had been published in the previous five years, and all (*n* = 7) had been published in the previous ten years. Four of the studies had been conducted in Europe (in Serbia, Norway, and two in the Netherlands), with two in the USA and one in Japan. Each study's specific objective is presented in Table 1.

The seven publications covered data from a total of 16668 people. The sample size was ranging from 1 to 14375. Among the 16668 people, 12649 were healthy controls, 3918 were cancer survivors, and 101 were ill cancer patients. With regard to treatment, 568 had received chemotherapy, 688 had received radiotherapy, 1799 had undergone surgery (some people had received two or more treatments), and one study did not specify the survivors' previous treatments [28]. The weighted mean age of cancer survivors and cancer patients was 77. More than half of the study participants were women (60%), although the samples were very heterogeneous: the proportion of women ranged from 0% to 100%. This could be explained by the wide range of types of cancer studied, some of which affect one sex solely or primarily (e.g., prostate cancer and breast cancer) (Table 2).

In accordance with our study objectives, we will describe the effects of cancer and its treatment on leisure, profiles of older adults with cancer who are restricted in their participation in leisure and productive occupations, and rehabilitation approaches with an impact on participation in leisure and productive occupations; we also added a part about cancer and physical activities in leisure or productivity, as it was an important result of our review.

### 3.3. Effect of Cancer and Its Treatment on Leisure

Two articles focused on leisure and/or socialization (one subdomain of leisure according to the CMOP-E). Fossa et al. [29] used a cross-sectional study (*n* = 612) to explore the effects of the typical adverse events of curative treatment for prostate cancer (prostatectomy vs. radiotherapy) on global quality of life in cancer survivors. There was one specific item on limitations of social life and/or leisure and another one on family life. The authors used a custom questionnaire with multiple-choice questions, questions about treatment outcomes, a free text field, a visual analogue scale, and the Expanded Prostate Cancer Composite-26 (EPIC-26). The patients' partners were also interviewed. Although the exact type of limitations of leisure or family life was not specified, 14% of the older adults having undergone prostatectomy and 18% of those having received radiotherapy (*p* = 0.001) reported limitations in their social life or leisure activities. Results showed that social life and/or leisure activities had a significant association with quality of life, even in a multivariate analysis (*p* = 0.001), whereas family life did not, for both patients having undergone prostatectomy or radiotherapy.

Berat et al. [30] focused their cohort study (*n* = 150) on social functioning in older adults with cancer in Serbia. In their study, the concept of social functioning was limited to visits and phone calls from friends, family, and relatives. So they assessed and compared the frequency of social contacts in older adults undergoing chemotherapy for early-stage carcinoma, older adults undergoing chemotherapy for advanced-stage cancer, and a control group of healthy older adults. The two groups of cancer patients were assessed just before the first cycle of chemotherapy and then three months afterwards. The authors found that cancer and its treatments initially led to more social contacts but then often contributed to the exclusion of people from their social environment—especially when the cancer had been diagnosed some time ago. Indeed, they found that the proportion of older adults who had received a visit from their relatives during the first treatment cycle (or, for healthy controls, over the same period) was 31% for both the early-stage and late-stage cancer groups and 16% in the control group, while three months later, it was 24% for the early-stage cancer group, 22% for the late-stage cancer group, and still 16% in the control group. In addition, respectively, 29%, 22%, and 10% of participants often received phone calls from relatives during the first treatment cycle (or at the time of the assessment, for the healthy group); three months later, the proportions were, respectively, 12%, 18%, and 10%. However, the authors are cautious about the possibility of extrapolating these results in other contexts as Serbian cultural context “denies suffering and death.”

### 3.4. Cancer and Physical Activities in Leisure or Productivity

Blair et al. [28], Buffart et al. [31], and van Nieuwenhuizen et al. [32] studied the link between cancer and physical activities (PAs) among cancer survivors—including leisure PAs and household PAs, whereas Blair et al. performed a prospective cohort study (*n* = 14375) and Buffart et al. (*n* = 1371) and van Nieuwenhuizen et al. (*n* = 116) performed a cross-sectional study. Blair et al. and Buffart et al. both used the SF-36 QoL (the Medical Outcomes Study Short Form-36 item survey) questionnaire to explore the link between PA and quality of life, whereas van Nieuwenhuizen et al. used the European Organization for Research and Treatment of Cancer quality of life questionnaire core module (EORTC QLQ-C30) and the head and neck module (EORTC HN35).

One study showed that colorectal cancer survivors reported spending an average of 19.1 ± 14.7 h/week on PAs, including gardening, housekeeping, walking, cycling, and sports [31].

More specifically, for the head and neck cancer survivors, 54% of the reported activities were household activities, 34% were leisure activities, and 12% were productive activities [32].

Finally, when comparing frequency between cancer-free and breast cancer survivor women over time, the proportion of people who stayed active between the first survey (1986) and the second survey (2004) was 38% for cancer-free women and 34% for cancer survivors. Furthermore, 19% of the cancer-free women and 17% of the cancer survivors became active, respectively, 18% and 22% became inactive, and 24% and 28% remained inactive. So overall, cancer-free women were more active than cancer survivors [28].

The three studies insisted on the importance of including all PAs performed by older adults—i.e., household activities and less vigorous activities—when seeking to accurately measure quality of life.

Accordingly, Lyons et al.'s [33] cross-sectional study (*n* = 43) explored changes in activity levels among older adult cancer survivors, by using the Activity Card Sort modified (ACSm) to assess changes over time from before the diagnosis of cancer to three months after the completion of cancer treatment in 80 activities. The ACSm score was then separated into four subscales: low-demand physical leisure, high-demand physical leisure, instrumental activities, and social activities. The Patient Health Questionnaire and the Comorbidity Index were also rated. There was a 12% reduction in overall activity, a 34% reduction in high-demand physical leisure, and a 16% reduction in social activities. However, even though many patients reduced their level of activity or stopped some activities completely, they also started some new activities (mostly low-demand activities, such as resting or spending more time with relatives). Furthermore, the study participants usually decided to put their energy into the activities that they most valued, regardless of the energy cost. As in Buffart et al.'s study, the changes were explained mainly not only by fatigue but also by appetite disturbance, changes in interest, and physical changes. Some participants even reported that they had voluntarily stopped or reduced activities they did not enjoy or had lowered their expectations of achieving some activities (such as not always having a clean house, for example): this highlights the need to take account of satisfaction when exploring participation in occupations. Finally, ACSm scores were positively associated with the quality of life, health, and functioning and were able to discriminate between healthy and ill populations. On the basis of these results, the authors insisted on the need for balance between rest and physically demanding activities during a typical day. However, the researchers pointed out that all the interviewees were being successfully treated for cancer and so may have had a different point of view than people with more comorbidities or a more life-threatening cancer.

### 3.5. Profiles of Older Adults with Cancer Who Are Restricted in Their Participation in Leisure and Productive Occupations

Buffart et al. [31] highlighted the link between fatigue and the SF-36 physical subscale and found that people with comorbidities were less active. Hence, older cancer patients with a high level of fatigue and/or more comorbidities are more at risk of low occupational participation. van Nieuwenhuizen et al. [32] extended this finding by showing that household activities accounted for an increasing proportion (30-60%) of the total daily PA in older adults. Furthermore, older head and neck cancer survivors were more at risk of low PA engagement.

In view of the results of these studies, we conclude that age, the level of fatigue, the level of PA before cancer, and comorbidities may influence engagement in leisure and productivity.

### 3.6. Rehabilitation Approaches with an Impact on Participation in Leisure and Productive Occupations

Rehabilitation approaches including occupational therapy were described in two articles.

In a case study of a 66-year-old woman with lung cancer and metastases in the lumbar vertebrae, Imanishi et al. [34] examined changes in the quality of life and the causes of those changes during in-home occupational therapy. However, as a case study, it had a low level of evidence. The researchers applied the Philadelphia Geriatric Centre Morale Scale and the 100-Point Satisfaction Scale (a simplified visual analogue scale for happiness). Four assessments were made before the woman died. However, the researchers detected an increase in the quality of life when they focused on the client's demands, using an approach based on the COPM: firstly, the client wanted to be able to use the toilet without assistance (i.e., self-care) and later wanted to write to her relatives (i.e., leisure) and take a trip to her home town (again leisure) against her physician's advice.

Similarly, Lyons et al. [33] also assessed occupational therapy tools in their study. They insisted on the importance of guiding the client into balancing physically demanding activities and rest during their recovery—even replacing occupational therapy sessions with relaxation when the occupational therapist felt that the client was fatigued (a factor also mentioned by Buffart et al. [31]). Lyons et al. emphasized the importance of assessing rest, leisure participation, and social participation when initiating occupational therapy.

### 3.7. Study Quality Assessment

Two studies were judged to be of fair quality on the NOS scale (i.e., “2 stars in selection domain AND 1 or 2 stars in comparability domain AND 2 or 3 stars in outcome/exposure domain”). Of the publications evaluated with the adapted NOS scale, 2 were judged to be of low quality and thus to have a high risk of bias. The remaining studies scored ≥10 on the adapted NOS (Table 2).

## 4. Discussion and Implications

As discussed previously, in healthy older adults, engaging in self-care occupations is important but so too is participation in leisure and productive occupations [15–17, 19, 20]. Cancer and its treatments can lead to functional limitations, especially among older adults [35], which can affect occupational engagement [36], especially as cancer can become a chronic disease [37], thus with long-term occupational disruptions. With view to the quality of life, it is thus important to offer the possibility for the client to express occupational problems in each of the three occupational domains and to be able to support those problems with evidence-based solutions. However, this systematic review about leisure and productivity among older adults with cancer did not reveal any article focused on this topic, except for Lyons et al.'s study of changes in the activities (including leisure and productivity) of older adults with cancer [33]. Imanishi et al. [34] also studied all kinds of occupation in an end-of-life context, but as this was a single case, it prevents extrapolating the results. Five other studies were analysed because they assessed some aspects of leisure and/or productivity (such as PAs and social functioning) in older adults with cancer. The main difficulty was that the few studies dedicated to occupations of older adults with cancer focused on the impact of participating in activities regarding health, rather than the impact of cancer and its treatment on participation and engagement in personally valued occupations notably leisure and productivity. However, previous studies have shown the importance of being able to continue routines and occupations, sometimes implanted many years ago, especially among older adults [38, 39]. Further, occupations are a strong determinant of the identity, and being able or not being able to engage in occupations changes the way the illness is experienced: the consequences of the illness on daily living rather than the illness itself can affect people living with cancer [36].

This low number of studies could be explained by the lack of rehabilitation with this population and especially occupational therapy: a recent scoping review showed the lack of literature regarding cancer care and occupational therapy [40], and this might be even more true for older adults [2, 41]. This can be dramatic for the clients, as it has been shown that unmet rehabilitation needs were related to a lower quality of life when struggling with cancer [42]. This can be especially relevant in the context of older adults, who might experience unique lifespan and developmental challenges, such as loss and bereavement, addition of grandchildren or great-grandchildren, new caregiving responsibilities, loss of function, retirement, and comorbidities, and might value daily rituals even more than younger adults [43] and thus be even more in need for occupation-centred rehabilitation.

Therefore, the results of this systematic review show the urgent need for occupational therapists to explore more in-depth the opportunities for clinical practice with this population and for researchers to show the needs and benefits of occupational therapy with this population. More specifically, little information has been published about ways of improving leisure and productivity among older adults with cancer. Imanishi et al. [34] found that occupational therapy was beneficial, although this was a single case study. Additionally, Lyons et al. [33] mentioned that occupational therapy should include the assessment of all types of occupations during patient follow-up. Those results are supported by several national association recommendations [21, 44, 45] and studies [40, 46] which highlight the role of occupational therapists to help people living with cancer to (re)-engage in valued occupations of all kind and the role of occupational therapy at each stage of the disease (from prevention till end-of-life care) [40, 43]. Thus, further evidence would be needed to assess the exact impact of occupational therapy on leisure and productivity in this population.

Assessment should also be updated because (i) symptoms such as fatigue and pain fluctuate and (ii) patterns change as a function of the time since cancer diagnosis. For example, women initially performed more PA but then performed less [28], and children and relatives were more present shortly after the diagnosis but then became less present [30]. The results of our review also suggest that both physical and emotional/psychological aspects should be taken into account in the assessment of leisure and productivity [31, 33]. Some studies focused on a single aspect of leisure and/or household activities, such as PAs. In this context, one can question the accuracy of the tools described in the reviewed publications for study leisure and/or household activities. For example, the SF-36 does contain items related to leisure but is restrictive if one wishes to study this aspect specifically. Furthermore, the tools were valid and reliable but featured closed questions; this can limit the picture of leisure and household PAs performed by older adults with or without cancer. It might be worth using occupation-centred tools such as the COPM, the ACS, or the Occupational Self-Assessment (OSA). This supports Larsson et al. stating that many studies regarding older adults only use ADL measures to assess disability, although it would be relevant to also include subjective assessment about what older adults themselves see as important [39].

Further, “social functioning” and “physical functioning” were not clearly described and thus often remained vague. Although van Nieuwenhuizen et al. [32] applied the Physical Activity Scale for the Elderly (PASE), they did not use the tool's open-ended options. By way of an example, Desmond et al.'s [47] study of 6503 respondents from the 2013 Behavioral Risk Factor Surveillance System (BFRSS) used open questions. This enabled the researchers to draw up a list of the Alabama residents' most common occupations, with a focus on the importance of assessing leisure and household activities when measuring PAs. Desmond et al. found that the main primary PAs among older (65+) participants (*n* = 2411) were walking (63%), gardening (13%), and cycling (3%) and showed that some PAs can be classified either as “vigorous” or “moderate,” depending on the person's age: the older you are, the more demanding a given PA becomes. Consequently, the expertise of occupational therapists about engagement in all types of occupations across the lifespan would be of great value. Among the studies reviewed here, only Lyons et al. [33] completed the ACSm with interviews in order to gain more in-depth knowledge of the client's occupations (including leisure and productivity).

Our systematic review also provided information about the profile of older adults whose participation in leisure and productivity might be affected. This especially includes older people with more comorbidities [28, 31] and with a high level of fatigue [31, 33, 34]. This is congruent with previous studies showing that cancer-related fatigue impacted daily routine in 88% of adults and that the fatigue symptom could have a functional impact on performance areas such as work, ADL and leisure, and health-related quality of life [48]. This highlights the need for a balance between activity and rest, such as suggested by [33, 48].

Lastly, only 2.6% of the total cancer population studied here (101 out of 3918) were being treated for cancer at the time of the assessment; the other people were survivors. Hence, there is a need for much more research on the impact of cancer on participation and engagement in leisure and productivity among actively treated older adults.

### 4.1. Limitations

Our systematic review had some limitations. Firstly, the concepts of leisure and productivity are broad, and it is difficult to be sure whether we detected all the published studies in these areas. For example, a publication about a single, specific type of leisure activity might not have been found by our search algorithm. Secondly, half of our publications were not specific for older adults [29, 31, 32], and none of those offered a specific analysis of results for the over 65s. Also, we only found a few articles exactly matching our criteria: this did highlight the dramatic need for further practice and research with this population, but it also prevented us from reaching any solid and transferable conclusions about the impact of cancer regarding leisure and productivity in older adults. Finally, our findings and subsequent conclusions have to be positioned from a predominantly Western perspective; as except one study from Japan, all other included studies were from either Europe or the USA.

### 4.2. Implication for Research

This systematic review showed that little is known about the impact of cancer and its treatments on participation and engagement in leisure and productivity among people aged 65 or more. However, several studies have shown that healthy older adults are highly interested in these kinds of occupation (e.g., [19, 42, 49, 50]). Hence, it will be important to gather as much evidence about the impact of cancer and its treatment on leisure and productivity among older adults as it has been gathered for self-care. According to the previous results, more research would also be needed to better determine the clinical and sociodemographic profile of older adults at risk of occupational disruptions. In view of their expertise in occupations, occupational therapists are ideally placed to conduct this type of research.

Hence, more research is warranted with regard to (i) the nature of this balance and (ii) the classification of activities as “active” or “quiet” for older adults with cancer as a consequence of interactions between their personal, environmental, and occupational characteristics. This would be the first step for research providing evidence of the impact of cancer on leisure and productivity, thereby demonstrating the best occupational therapy practice for this population.

### 4.3. Implication for Practice

Our systematic review showed that cancer and its treatment can lead to a reduction in engagement in occupations. It is essential for the client to choose which occupations he/she wishes to continue, to adapt, or to abandon, related to energy-management balance [33]. Given the above results, we consider that it is important to manage the balance between active and quiet activities, as suggested by Lyons et al. [33]. This would allow clients to decide in which very valued occupation they prefer to put their energy to continue engaging in the occupations defining their selves [36], using energy conservation techniques which have been found to be successful in reducing cancer-related fatigue [48]. Furthermore, some “moderate” activities become “vigorous” with age [47], so there is a need to carefully assess the energy level of a given occupation for a given person. Occupational therapists may help with this choice by assessing functional and occupational problems of older adults at risk of participation limitations [51]. The use of occupation-centred and client-centred specific tools (e.g., the COPM, the ACS or ACSm, and the OSA) may help to achieve this goal [52, 53]. Taking both physical and emotional aspects into account is also essential [31, 33]. Lastly, therapists should also consider including the patient's relatives in the assessment of occupations and in the definition of rehabilitation goals as this would allow inclusion of cooccupations as seen in this study [54]. Regarding all those results, occupational therapists could also help to better identify older people with cancer at risk of occupational disruption. Those points are all considered basics for occupational therapists in general; however, it seems underused with this specific population.

## 5. Conclusion

Although there are a significant number of publications regarding the impact of cancer on function and personal care, there are few publications offering an occupation-centred approach about the impact of cancer and its treatments on leisure and productivity among older adults with cancer or survivors. However, age, the level of fatigue, the level of physical activity before cancer, and comorbidities may be risk factors for reduced engagement in occupations of all kind. Considering the link between occupational engagement and health and well-being, it seems important to go beyond the functional approach, into an occupation- and client-centred approach in occupational therapy.

## Figures and Tables

**Table 1:** Stated aims of the included studies.

First author Year	Stated aim
Berat, S. 2015	To determinate social functioning of elderly people suffering from malignant diseases and the possibilities for their social integration
Blair, C.K. 2016	Examining the degree to which physical inactivity is associated with poor QoL among older, long-term female cancer survivors compared to similar-aged women without cancer
Buffart, L.M. 2012	To describe the physical activity level in a large group of Dutch colorectal cancer survivors and to identify which demographic and cancer-related factors were associated with physical activity; the second aim was to study whether physical activity was associated with health-related quality of life and whether this association was medicated by fatigue and distress
Fosså, S.D. 2015	To explore the effect of typical adverse effects on global quality of life, if analysed together with other medical and psychosocial health conditions as reported by prostate cancer patients who considered themselves tumour-free after curatively intended treatment. Perception of the quality of their partnership was explored as a secondary aim
Imanishi, M. 2015	To examine the application of occupational therapy in the final stage of life by following the path of a patient who transitioned from denial of disease and death to acceptance and desire to live their remaining life to the fullest
Lyons, K.D. 2013	To explore survivors' activity levels 3 months after completion of cancer treatment
Van Nieuwenhuizen, A.J. 2018	To describe the level of physical activity among head and neck cancer survivors, including leisure time, household, and occupational physical activities; to study demographic, clinical, and lifestyle-related correlates of physical activities; and to assess the association between physical activities and health-related quality of life adjusted for important demographic, clinical, and lifestyle-related factors

**Table 2:** Characteristics of the included studies.

Authors Year	Title	Journal	Country of the study	Sample size (*n*)	Age, mean ± SD (years) [range]	Male/female (%)	Cancer site/type (% of the total sample)	Study design (level of evidence)	NOS^∗^ or adapted NOS score: number of stars given
Berat, S., Nešković-Konstantinović, Z., Nedović, G., Rapaić, D., Marinković, D. 2015	Social Functioning of Elderly Persons with Malignant Disease	Vojnosanitetski Pregled	Serbia	150	70.39 ± 4.29 [65-79]	19/81	Healthy (33.33) Breast cancer (29.33) Colorectal cancer (12.67) Gynaecological cancer (10.00) Other (14.67)	Exposed/nonexposed prospective cohort study (III)	NOS: 7 out of 9

Blair, C.K., Robien, K., Inoue-Choi, M., Rahn, W., Lazovih, D.A. 2016	Physical Inactivity and Risk of Poor Quality of Life among Elderly Cancer Survivors Compared to Women Without Cancer: The Iowa Women's Health Study	J Cancer Surviv.	USA	14375	78.6 ± 3.9 [73-88]	0/100	Healthy (87.66) Breast (5.76) Colorectal (2.25) Gynaecologic (1.69) Melanoma (0.62) Urinary (0.56) Haematological (0.56) Short survival (0.27) Other (0.63)	Exposed/nonexposed prospective cohort study Auxiliary study (III)	NOS: 7 out of 9

Buffart, L.M., Thong M.S.Y., Schep, G., Chinapaw, M.J.M., Brug, J., Van de Poll-Franse, L.V. 2012	Self-Reported Physical Activity: Its Correlates and Relationship with Health-Related Quality of Life in a Large Cohort of Colorectal Cancer Survivors	PLoS ONE	The Netherlands	1371	69.5 ± 9.7 [*Not reported*]	56/44	Colon (66.23) Rectal (33.77)	Cross-sectional study (IV)	Adapted NOS: 13 out of 16

Fosså, S.D., Dahl, A.A. 2015	Global Quality of Life after Curative Treatment for Prostate Cancer: What Matters? A Study among Members of the Norwegian Prostate Cancer Patient Association	Clinical Genitourinary Cancer	Norway	612	69.00 (SD not reported) [47-105]	100/0	Prostate (100.00)	Cross-sectional study (IV)	Adapted NOS: 5 out of 16

Imanishi, M., Tomohisa, H., Higaki, K. 2015	In-Home Occupational Therapy for a Patient with Stage IV Lung Cancer: Changes in Quality of Life and Analysis of Causes	SpringerPlus	Japan	1	66.00 (**±**0) [66-66]	0/100	Lung (100.00)	Report case study (V)	Adapted NOS: 4 out of 16

Lyons, K.D., Lambert, L.A., Bala, S., Hegel, M.T., Bartels, S. 2013	Changes in Activity Levels of Older Adult Cancer Survivors	OTJR	USA	43	72.00 (**±**8) [60-over 90]	44/56	Breast (34.88) Gastrointestinal (27.91) Haematological (13.95) Genitourinary (13.95) Lung (4.65) Head and neck (4.65)	Cross-sectional study (IV)	Adapted NOS: 10 out of 16

Van Nieuwenhuizen, A.J., Buffart, M., Van Uden-Kraan, C.F., Van der Velden, L.A., Lacko, M., Brug, J., Leemans, C.R., Verdonck-de Leeuw, I.M. 2018	Patient-Reported Physical Activity and the Association with Health-Related Quality of Life in Head and Neck Cancer Survivors	Support Cancer Care	The Netherlands	116	60.00 (±10) [*Not reported*]	63/37	Oral cavity and oropharynx (48.28) Larynx and hypopharynx (28.45) Other (23.27)	Cross-sectional study (IV)	Adapted NOS: 10 out of 16

^∗^Newcastle–Ottawa Scale.

## Data Availability

The data used to support the findings of this study are available from the corresponding author upon request.
